# Medical College Notes

**Published:** 1891-06

**Authors:** 


### Medical College Notes.

THE UNIVERSITY OF BUFFALO.—The following changes and additions have been made to the teaching staff of the Medical Department:

Dr. P. W. Van Peyma, Adjunct Professor of Obstetrics; Dr. De Lancey Rochester, Adjunct Professor of the Principles and Practice of Medicine; Dr. Eli H. Long, Adjunct Professor of Materia, Medica and Therapeutics; Dr. Wm. H. Bergtold, Adjunct Professor of Pathology; Dr. James W. Putnam, Professor of Diseases of the Nervous System. Dr. F. Whitehill Hinkel, Professor of Laryngology; Dr. Frank H. Potter, Clinical Professor of Laryngology; Dr. Wm. H. Heath, Associate Professor of Genito-Urinary and Venereal Diseases; Dr. Herbert U. Williams, Lecturer on Biology; Dr. Fred B. Willard, Instructor in Anatomy; Dr. Allen A. Jones, Instructor in Medicine; Dr. A. L. Benedict, Instructor in Materia Medica and Therapeutics; Dr. M. A. Crockett, Instructor in Obstetrics and Gynecology; Dr. Wm. H. Chace, Instructor in Chemistry. Other appointments are soon to be made.

The Medical Department will soon occupy more commodious buildings. The Council of the University has bought a large lot on High street, upon which a handsome college edifice is to be erected at a cost of not less than $125,000; the term is to be extended to seven months; the course graded, and every department fully equipped with teachers. Dr. John Parmenter has succeeded Dr. Chas. Cary as Secretary and Treasurer of the Faculty.

